# Gene Polymorphism of MUC15, MMP14, BRAF, and COL1A1 Is Associated with Capsule Formation in Hepatocellular Carcinoma

**DOI:** 10.1155/2021/9990305

**Published:** 2021-04-28

**Authors:** Wei Sun, Yongchao Zhang, Bozhi Liu, Youjia Duan, Wei Li, Jinglong Chen

**Affiliations:** Cancer Center, Beijing Ditan Hospital, Capital Medical University, Beijing, China

## Abstract

**Background:**

The presence of a capsule is an important prognostic factor in hepatocellular carcinoma (HCC). Capsule formation is affected by tumor-host interaction, which may include collagen deposition and extracellular matrix (ECM) degradation.

**Purpose:**

This study aimed to examine whether single-nucleotide polymorphisms (SNPs) in the genes for COL1A1 MUC15, MMP14, CD97, SMYD3, BRAF, and transforming growth factor beta 1 (TGF-*β*) are related to capsule formation.

**Methods:**

We prospectively recruited and analyzed 185 patients with HCC with or without a capsule between 2019 and 2020. The SNPs involved were analyzed by polymerase chain reaction. Differences in the allele and genotype frequency between the cases and controls were evaluated using the chi-square test. Odds ratios and 95% confidence intervals were calculated by logistic regression analysis with adjustment for age and sex. Stratification analyses were also performed with preselected variables.

**Results:**

The single-locus analysis showed that the presence of a capsule was significantly associated with five SNPs : MUC15 rs17309195 (*P*=0.01), rs12271124 (*P*= 0.02), rs10430847 (*P*=0.04), MMP14 rs17884816 (*P*=0.01), and BRAF rs74512895 (*P*=0.03). Adjusted logistic regression revealed that the decreased capsule formation was statistically significantly associated with BRAF rs76603725, COL1A1 rs2269336, and MUC15 rs17309195, while MMP14 rs17884816 and MUC15 rs10430847, rs2063278, and rs967490 were associated with increased capsule formation. The MUC15 block 2 haplotype was associated with increased capsule formation.

**Conclusions:**

MUC15, MMP14, BRAF, and COL1A1 gene polymorphisms are associated with capsule formation in HCC. Studies involving larger samples are needed to confirm our results.

## 1. Introduction

Worldwide, hepatocellular carcinoma (HCC) is the sixth most common cancer, and its incidence in the United States is increasing [1]. Although HCC may be curable by resection, liver transplantation, or ablation at the early stage and transarterial chemoembolization and systematic therapy can improve survival in unresectable HCC, most patients have poor prognosis due to recurrence, progression, or metastasis after therapy. Notably, the presence or absence of a capsule surrounding the tumor is closely associated with disease progression and long-term survival [2–5].

Pathologic or radiological examination has revealed a capsule around the tumor in 10–76% of patients with HCC [6, 7]. Although the underlying mechanism for capsule formation is unclear, a few studies have reported that the capsule consists mainly of type I and III collagen fibers and that the type I collagen mainly consists of collagen type I *α*1 (COL1A1) [8–10]. It is well known that stellate cells play a pivotal role in the development of liver fibrosis. Activated hepatic stellate cells (HSCs), termed myofibroblast-like cells, that express the *α* isotype of smooth muscle actin (*α*-SMA) are the principal source of capsule [8].

Matrix metalloproteinases (MMPs) are a family of enzymes that degrade the extracellular matrix (ECM), basement membrane, and growth factors, promoting tumor invasion and metastasis [11, 12]. MMP2 is one of the most studied MMPs, whose families are overexpressed in liver cancer. [13] The association between MMP2 and capsule has been investigated [14–16]. Moreover, the mucin 15- (MUC15-) and MT1-MMP- (MMP14-) encoding genes are significantly related to capsule formation in liver cancer through the regulation of MMP2 expression. Patients with capsule have lower MUC15 expression than patients without capsule due to the regulation of MMP2 expression levels in the liver through the PI3K-AKT signaling pathway [17]. Similarly, MT1-MMP is an essential substance in MMP2 activation and is highly expressed in the periphery of the tumor [14]. MT1-MMP expression levels are significantly different between patients with liver cancer with intact and incomplete capsules [18]. Further, a cluster of differentiation 97 (CD97) and SMYD3, a member of the SET and MYND domain (SMYD) family, have been associated with the presence of capsule via regulation of MMP2. CD97 regulates MMP2 expression via G protein-coupled receptor 6 [19]. Moreover, the SMYD3 gene increases MMP2 expression levels in the liver by binding to the MMP2 promoter region and increasing H3K4me3 modification to promote gene transcription [20].

Chronic hepatitis B virus (HBV) infection promotes liver cirrhosis and results in HCC, particularly in China. The HBV X protein (HBx) affects fibrosis in the development of cirrhosis via transforming growth factor beta (TGF-*β*), which activates HSCs [21]. Consequently, TGF-*β* may modify capsule formation. In addition, BRAF expression is associated with the increased capsule formation in other tumors and has yet to be explored in HCC [22–24].

The presence of capsule has been proven to be a prognostic factor in patients with HCC. While several studies have revealed that the potential mechanism in capsule formation is associated with collagen fiber deposition and ECM degradation, the single-nucleotide polymorphisms (SNPs) in the related genes above remain to be elucidated. Furthermore, no SNP studies have reported the association of specific genes with capsule formation. Therefore, we conducted the present study to investigate the association between SNPs in genes affecting capsule formation and patients with HCC with or without capsule.

## 2. Materials and Methods

### 2.1. Study Participants

The study was conducted in line with the Declaration of Helsinki and the use of peripheral blood samples and clinical data was approved by the Institutional review board at the Beijing Ditan Hospital (approval number: 2019-067-01). Written informed consent was obtained from the patients. From May 2019 to January 2020, primary HCC were recruited prospectively. The criteria of inclusion included (i) patients with HCC confirmed by pathological or radiological examination for the first time, (ii) age between 18 and 75 years, (iii) HBsAg (+) >6 months, (iv) life expectance ≥3 months, (v) an Eastern Cooperative Oncology Group performance status of 0 to 1, (vi) tolerance to received CT or MRI examination, and (vii) life expectance ≥3 months. The criteria of exclusion included (i) life expectance ≤3 months, (ii) other invasive malignant, and (iii) intolerance to received CT or MRI examination.

Basic clinical information of the group was collected, including age, sex, HBsAg, HBV DNA, BCLC stage, AFP, tumor size, number of tumors, tumor location, vascular invasion, extrahepatic metastasis, other indicators including alanine aminotransferase, alanine aminotransferase, total bilirubin, and albumin. This study was conducted after obtaining informed consent from each participant and following ethical standards.

### 2.2. Tag SNP Selection

Tag SNPs were selected by employing genotype data from the International HapMap Project (http://hapmap.ncbi.nlm.nih.gov) data. A set of tSNPs which estimated *r*^2^ > 0.8 were defined compared with those untyped SNPs. The SNPs that have a minor allele frequency >0.05 in Chinese Han people by the Haploview 4.2 program (http://www.broad.mit.edu/haploview/haploview-downloads) were selected. Therefore, a total of 85 SNPs were identified in our study.

### 2.3. SNP Genotyping Assays

The iPLEX chemistry on a matrix-assisted laser desorption/ionization time-of-flight mass spectrometer (Sequenom, Inc.) was used to type SNPs. Polymerase chain reaction (PCR) was performed as previously described [25]. Laboratory staff are blind to patient information during genotyping assays.

### 2.4. Statistical Analyses

Differences in frequencies of the alleles and genotypes between the cases and controls were assessed by the chi-square test. Hardy–Weinberg equilibrium (HWE) in control subjects was assessed by the chi-square test for each SNP. Odds ratios (ORs) and 95% confidence intervals (CIs) were evaluated by logistic regression with adjustment for age and sex. Stratification analyses were also performed by related variables, such as age, sex, liver cirrhosis, tumor characteristics, extra-hepatic metastasis, and vascular invasion. SPSS 26.0 software (SPSS Inc., Chicago, Illinois, USA) was used to conduct data analyses.

Among the SNPs, Lewontin's standardized coefficient D' and LD coefficient *r*^2^ were used for the pairwise linkage disequilibrium (LD) and haplotype blocks defined in Haploview 4.2 with default settings.

## 3. Results

### 3.1. Patient Characteristics

A total of 185 cases (149 men and 36 women) were enrolled in this study. The age range was 31–83 years, and the average age was 59.02 ± 10.57 years. There were 89 patients in the capsule group, and 96 patients in the noncapsule group, which formed the control group. Overall, the cases and controls were age- and sex-matched well (*P*=0.586 and *P*=0.319, resp.). Similarly, other variables in the case and control groups were well-matched except for the Child–Pugh class.  Table 1 shows the detailed baseline characteristics of the patients. 14 patients in the capsule group and 8 patients in the noncapsule group are no cirrhosis. We further evaluated fibrosis-4 index (FIB-4) as a noninvasive indicator of staging of fibrosis in these patients, owing to aspartate aminotransferase to platelet ratio index (APRI) inferior to FIB-4 in CHB patients. FIB-4 scores are 0.93 ± 0.69 in the capsule group and 1.15 ± 0.74 in the noncapsule group, respectively (*P* > 0.05), which indicates that no significant fibrosis (F2) exists in these patients [26].

### 3.2. Individual SNP Association Analysis

The genotyping results for the quality control showed 97–99.7%. Supplementary Table S1 lists the SNP IDs, locations, and allele frequencies. In the 85 selected SNPs, the genotype distributions of all SNPs in the noncapsule group were consistent with those expected from HWE (all, *P* > 0.05) (Supplementary Table S1). The single-locus analyses showed that the allele frequencies of five SNPs were significantly different between the capsule group and the noncapsule group: BRAF rs74512895: *A* > *G*(*P*=0.03); MMP14 rs17884816: *T* > *C*, *G*(*P*=0.01); and MUC15 rs17309195: *G* > *A*(*P*=0.01), rs12271124: *C* > *T*(*P*=0.02), rs10430847: *T* > *A*(*P*=0.04). In addition, there was marginal evidence for COL1A1 rs2269336: *G* > *A*(*P*=0.05), MUC15 rs11822751: *C* > *T*(*P*=0.05), BRAF rs76603725: *T* > *C*(*P*=0.08), and MMP14 rs11622371: *T* > *C*(*P*=0.08).  Table 2 summarizes the differences in the genotype distributions of SNPs in the cases and controls.

**Figure 1:**
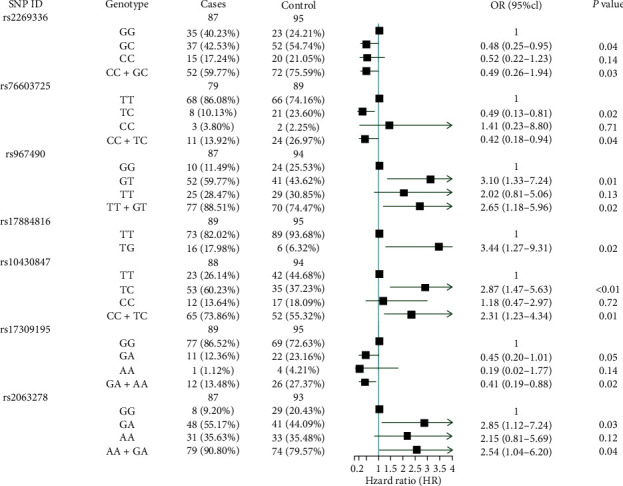
Logistic regression analysis of associations between the genotypes of selected SNPs and capsules formation.

Figure 1 shows that further logistic regression analyses adjusted for sex and age showed that, compared with wild-type carriers, decreased chance of capsule formation was associated with COL1A1 rs2269336 genotypes (GC/GG: adjusted odds ratio [OR] = 0.48, 95% confidence interval [CI] = 0.25–0.95, *P*=0.04), and BRAF rs76603725 genotypes (TC/TT: adjusted OR = 0.32, 95% CI = 0.13–0.81 *P*=0.02), but elevated capsules formation was significantly associated with MUC15 rs967490 genotypes (GT/GG: adjusted OR = 3.10, 95% CI = 1.33–7.24, *P*=0.01), rs10430847 genotypes (TG/TT: adjusted OR = 2.87, 95% CI = 1.47–5.62, *P*=0.02), rs2063278 genotypes (GA/GG: adjusted OR = 2.85, 95%CI = 1.12–7.24, *P*=0.03); and MMP14 rs17884816 genotypes (TG/TT: adjusted OR = 3.44, 95% CI = 1.27–9.31, *P*=0.02).

We further evaluated the associations of the SNP variant genotypes with capsule formation stratified by selected variables (Supplementary Table S2). Compared with the common homozygous genotype, the effect of the GC or GC + CC variant genotypes for COL1A1 rs2269336 and the TC or TC + CC genotypes for BRAF rs76603725 was more evident in males, liver cirrhosis, tumor number ≥3, tumor maximum diameter ≥5 cm, and tumor in two lobes. Notably, COL1A1 rs2269336 variant genotypes were also significantly associated with HBV DNA <100 and extrahepatic metastasis, while BRAF rs76603725 genotypes were significantly associated with vascular invasion. Interestingly, MMP14 rs17884816 TG genotypes were associated with tumor maximum diameters, tumor in a single lobe, and absence of extrahepatic metastasis, except for the variables mentioned above, including male sex and liver cirrhosis.

The MUC15 rs10430847 variant genotypes were significantly associated with high capsule formation in nearly all subgroups in addition to subgroups of females, absence of liver cirrhosis, and presence of vascular invasion. Notably, apart from the male sex, HBV DNA <100, liver cirrhosis, and tumor number ≥3, the increased capsule associated with the rs967490 TT or TT + GT variant genotypes was also pronounced in tumor maximum diameter <5 cm, single lobe involvement, absence of extrahepatic metastasis, and absence of vascular invasion.

### 3.3. Haplotype Block Structure and Linkage Disequilibrium (LD) Analysis

**Figure 2:**
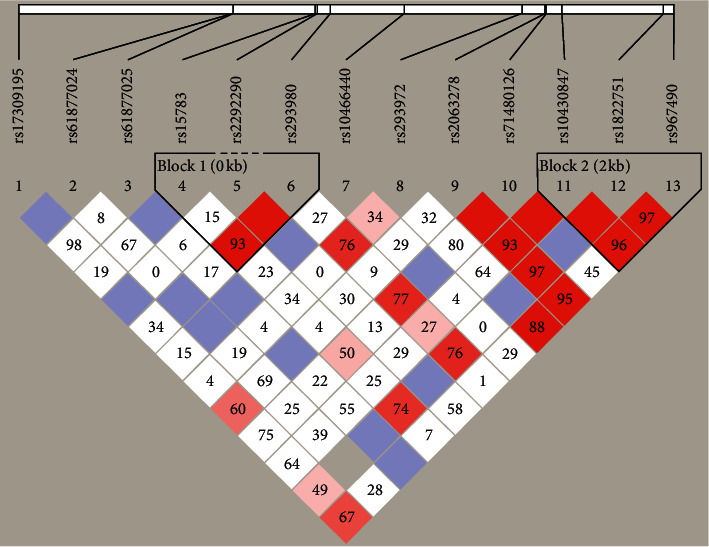
Graphical representation of the SNP locations and LD structure of MUC15. The SNP distribution and haplotype block structure across the MUC15 genes are shown, respectively. The measure of LD (*D*^2^) among all possible pairs of SNPs is shown graphically according to the shade of color, where white represents very low *D*^2^ and dark represents very high *D*^*2*^.

Figure 2 shows plots of the pairwise LD (D') values for the tSNPs and LD structures of the genes. The LD plot shows that there were two blocks with high LD : MUC15: block 1 (rs15783, rs29380) and block 2 (rs10430847, rs11822751, rs967490); for TGFB1, we identified the following region of strong LD: block 1 (rs1800469, rs4803457, rs2317130); for TGFB2, we identified the following regions of strong LD: block 1 (rs6691070, rs12058014, rs6604604, rs7550232, rs10482718) and block 2 (rs900, rs991967, rs3737977, rs6704255); for CD97, we identified two regions of strong LD: block 1 (rs62122578, rs9917022, rs10418487, rs10421249) and block 2 (rs3826759, rs12973667); for MMP14, we identified two regions of strong LD: block 1 (rs1003349, rs2269213) and block 2 (rs2236306, rs2236307). However, no block was found in the BRAF and COL1A1 genes. Among the blocks, there was a significant difference in MUC15 block 2 (TTG) between the cases and controls (*P*=0.03). Moreover, there was marginal evidence in the block of the TGFB1 gene (*P*=0.08) (Supplementary Figure).

## 4. Discussion

In this study, we examined the association between the presence of capsule and SNPs in the COL1A1, MUC15, MMP14, BRAF, TGFB1, and TGFB2 genes that might affect capsule formation in patients with HCC. Our results reveal that, of the selected tSNPs, five SNPs had significantly different allele frequencies between the cases and the controls: BRAF rs74512895; MMP14 rs17884816; and MUC15 rs17309195, rs12271124, and rs10430847. There was a significant difference in the genotype distributions of three SNPs : MMP14 rs17884816, MUC15 rs17884816, and rs10430847. Moreover, the haplotype analysis showed significant differences in the haplotype profile for MUC15 block 2. To the best of our knowledge, this is the first study to investigate a relationship between MUC15 and MMP14 SNPs and the presence of a capsule.

The presence or absence of a capsule is an important factor for tumor progression and prognosis. [4, 5, 27] The exact mechanism of capsule formation around tumors is not fully elucidated. It is believed that capsule formation is a result of tumor-host interaction. [10] Ooi et al. [8] and Ishizaki et al. [10] discovered that activated HSCs expressing *α*-SMA are responsible for tumor capsule formation in HCC patients with and without cirrhosis and metastasis stroma development, influencing local hepatic invasion. In addition, MMPs affect fibrogenesis in the capsule by degrading ECM proteins [13].

The ECM is a complex mainly composed of collagens, and type I collagen is one of the components of hepatic fibrosis [28]. COL1A1 gene polymorphism might be associated with liver fibrogenesis, and reduced COL1A1 mRNA expression has been significantly correlated with capsule formation in patients with HCC [29, 30]. Our findings that COL1A1 rs2269336GC genotypes were significantly associated with increased capsule formation in HCC are consistent with previous findings and further demonstrate that a capsule results from collagen deposition.

In tumors, MUC15 has been associated with lack of encapsulation by regulating MMP2 expression by blocking PI3K-AKT signaling [17]. While a number of studies have reported that MUC15 is involved in cancer development and influences cellular growth, invasion, and metastasis as a tumor suppressor, no MUC15 polymorphism has been examined in tumor association studies [31]. In the present study, we first demonstrated that several MUC15 SNPs were associated with capsule development in Chinese populations. Moreover, CD97 and SMYD3 promote tumor progression by regulating MMP2. However, we found no evidence of an association of variants in these two genes with encapsulation. These negative results might be partially due to the small sample size in our study. Further study of CD97 and SMYD3 function in capsule formation is warranted.

MMP14 is a membrane-bound collagenase and is the main activator of MMP2. MMP14 is used in the tumor microenvironment and promotes tumor invasion and metastasis. MMP14 polymorphisms influence cancer susceptibility and clinical characteristics, including that of cervical cancer, esophageal squamous cell carcinoma, and HCC [32–34]. Here, we demonstrate that the TG genotype of MMP14 rs17884816 was significantly associated with an increased risk of capsule formation. Several studies have demonstrated that low MMP14 expression was increased in encapsulated tumors compared with nonencapsulated tumors, which our results confirm [15, 35].

BRAF mutations have been significantly associated with capsule in papillary thyroid carcinomas, and BRAF signaling plays a critical role in regulating HCC cell proliferation [22–24]. Our results reveal that the TC genotype in rs76603725 was associated with a low risk of capsule formation. To date, this is the first study demonstrating a BRAF SNP associated with capsule formation.

HBV infection is a major cause of HCC in China. Martin-Vilchez et al. reported that HBx promotes liver fibrosis by activating HSC proliferation through the TGF-*β* pathway [21]. However, our study suggests that no TGFB SNPs are associated with an increased risk of capsule formation. Subgroup analysis in two meta-analyses has demonstrated that both HBV patients and Asian populations were not significantly associated with TGFB1 SNPs [36, 37].

Notably, the Child–Pugh B class is significantly different between the capsule group and noncapsule group in baseline characteristics of patients. This may be partly explained by the absence of capsule associated with more aggressive properties in HCC in that the presence of capsule might inhibit tumor cells' invasion, therefore exacerbating liver function [38]. Although there are a few limitations, the Child–Pugh class remains the most widely used grade to assess liver function and an independent prognostic factor for HCC patients. Selection of the most appropriate treatment in HCC patients with Child–Pugh B should be cautious. Most treatments in HCC patients with Child–Pugh B may counteract benefits by deteriorating liver function deserve [39]. The most suitable treatments for Child–Pugh B patients should be discussed in a multidisciplinary setting and consider patient intentions after an adequate assessment of potential risks and benefits.

Multiple tumors with no capsule in HCC patients are associated with poor survival after TACE [40, 41], while improved survival was observed in patients who received sorafenib or chemotherapy such as capecitabine [41–44]. Capsule formation related SNPs as a prognostic marker for a preferable treatment decision deserved further investigation in future studies.

Despite the fact that we prospectively enrolled patients with HCC, our study has limitations. First, the sample size is too small for underestimating potential SNPs associated with capsule formation, resulting in false positive results. Second, our results may overestimate the true effect size or identify spurious associations due to possible population stratification due to rare patients in certain subgroups. Moreover, there is selection bias in our study. The study population was of homogenous ethnic background (Chinese) and virus infection (HBV). Our results, therefore, warrant further investigation in other races and HCV infection-related patients with HCC.

## 5. Conclusions

To our knowledge, our study is the first to reveal that several SNP variants in the MUC15, MMP14, COL1A1, and BRAF genes might modulate the risk of capsule formation in patients with HCC. Overall, COL1A1 and BRAF SNPs are associated with a decreased chance of capsule formation, while MUC15 and MMP14 variants are associated with an increased chance of capsule formation. In particular, the MUC15 haplotype is significantly different between cases and controls. Studies with larger sample sizes are warranted to confirm our results.

## Figures and Tables

**Table 1:** Characteristics of patients.

Variable	Capsule (*n* = 89)		Noncapsule (*n* = 96)		*P* value
Age (years), mean ± SD	59.47 ± 10.425		58.56 ± 10.602		0.586

Sex, *n* (%)					
Male	69	77.50%	80	83.30%	0.319
Female	20	22.50%	16	16.70%	
HBsAg					
>250	53	59.60%	49	51.00%	0.243
≤250	36	40.40%	47	49.00%	

HBV DNA (copies), *n* (%)					
≥1000	15	16.90%	12	13.00%	0.402
＜1000	74	83.10%	84	88.00%	

Liver cirrhosis, *n* (%)					
Yes	75	84.30%	88	91.67%	0.12
No	14	15.70%	8	8.96%	

Child–Pugh class, *n* (%)					
A	89	89.89%	72	75.00%	0.024
B	8	8.99%	23	24.00%	
C	1	1.12%	1	1.00%	
ALT (IU/L), *n* (%)					
≥40	24	27.00%	32	33.30%	0.346
＜40	65	73.00%	64	66.70%	
AST (IU/L), *n* (%)					
≥40	30	33.70%	32	33.30%	0.957
＜40	59	66.30%	64	66.70%	
Tbil (mg/dL), *n* (%)					0.407
≥17.1	11	12.36%	16	16.67%	
＜17.1	78	87.64%	80	83.33%	
ALB					0.072
≥40	24	25.30%	20	32.60%	
＜40	71	74.70%	69	67.40%	

Tumor number, *n* (%)		89			0.552
1	47	52.81%	43	44.79%	
2	11	12.36%	14	14.58%	
≥3	31	34.83%	39	40.63%	

Tumor max diameters (mm), *n* (%)					0.182
≤5	47	52.81%	60	62.50%	
＞5	42	47.19%	36	37.50%	

Tumor location, *n* (%)					0.884
Left lobe	11	12.36%	12	12.50%	
Right lobe	45	50.56%	46	47.92%	
Both	31	34.83%	37	38.54%	
S1	2	2.25%	1	1.04%	

BCLC stage, *n* (%)					0.35
0	6	6.74%	13	13.54%	
A	24	26.97%	16	16.67%	
B	28	31.46%	31	32.29%	
C	30	33.71%	35	36.46%	
D	1	1.12%	1	1.04%	

AFP (ng/mL), *n* (%)					0.81
≤400	61	70.93%	63	69.23%	
＞400	25	29.07%	28	30.77%	
Extra-hepatic metastasis, *n* (%)		89			0.68
No	78	87.64%	86	89.58%	
Yes	11	12.36%	10	10.42%	

Vascular invasion, *n* (%)					0.31
No	68	76.40%	67	69.79%	
yes	21	23.60%	29	30.21%	
PVTT, *n* (%)	89		96		0.312
No	68	76.40%	67	69.79%	
Yes	21	23.60%	29	30.21%	
HVTT, *n* (%)					0.892
No	83	93.26%	90	93.75%	
Yes	6	6.74%	6	6.25%	

SD: stand deviation, AFP: alpha-fetoprotein levels, Alb: albumin, AST: aspartate aminotransferase, AL: alanine aminotransferase, T Bil: total bilirubin, PVTT: portal vein tumor thrombosis, HVTT: hepatic vein tumor thrombosis.

**Table 2:** Genotypes of selected SNPs and capsule formation.

SNP ID	Genotype	Cases		Controls		*P* value*∗*
rs2269336	Total	87	%	95	%	
	GG	35	40.23%	23	24.21%	0.068
	GC	37	42.53%	52	54.74%	
	CC	15	17.24%	20	21.05%	
	CC + GC	52	59.77%	72c	75.79%	

rs76603725		79		89		
	TT	68	86.08%	66	74.16%	0.065
	TC	8	10.13%	21	23.60%	
	CC	3	3.80%	2	2.25%	
	CC + TC	11	13.92%	24	26.97%	

rs17884816		89		95		0.015
	TT	73	82.02%	89	93.68%	
	TG	16	17.98%	6	6.32%	

rs10430847		88		94		0.007
	TT	23	26.14%	42	44.68%	
	TC	53	60.23%	35	37.23%	
	CC	12	13.64%	17	18.09%	
	CC + TC	65	73.86%	52	55.32%	

rs967490		87		94		0.029
	GG	10	11.49%	24	25.53%	
	GT	52	59.77%	41	43.62%	
	TT	25	28.74%	29	30.85%	
	TT + GT	77	88.51%	70	74.47%	

rs2063278		87		93		0.086
	GG	8	9.20%	19	20.43%	
	GA	48	55.17%	41	44.09%	
	AA	31	35.63%	33	35.48%	
	AA + GA	79	90.80%	74	79.57%	

∗Chi-squared test.

## Data Availability

The data used to support the findings of this study are included within the supplementary information files.

## Supplementary Materials

Supplementary Materials Supplementary Table S1 lists the SNP IDs, locations, and allele frequencies. Supplementary Table S2 shows that we evaluated the associations of the SNP variant genotypes with capsule formation stratified by selected variables. Supplementary Figure exhibits haplotype blocks of specific genes.
