# Supplementary material for: Gene Polymorphism of MUC15, MMP14, BRAF, and COL1A1 Is Associated with Capsule Formation in Hepatocellular Carcinoma
Source: Can J Gastroenterol Hepatol. 2021 Apr 28;2021:9990305. doi: 10.1155/2021/9990305 (PMC8100414; doi:10.1155/2021/9990305)
Supplement: Supplementary Materials — Supplementary Table S1 lists the SNP IDs, locations, and allele frequencies. Supplementary Table S2 shows that we evaluated the associations of the SNP variant genotypes with capsule formation stratified by selected variables. Supplementary Figure exhibits haplotype blocks of specific genes. [file 9990305.f1.zip › 9990305.f1/Supplementary Table S1.DOCX]

| SNP | Location | Gene | P for HWE | P for allele frequencies | P for genotypes |
| --- | --- | --- | --- | --- | --- |
| rs10404878 | 14395314 | CD97 | 0.17 | 0.79 | 0.62 |
| rs10412108 | 14397411 | CD97 | 0.52 | 0.37 | 0.36 |
| rs10416187 | 14395515 | CD97 | 0.70 | 0.26 | 0.49 |
| rs10418487 | 14384622 | CD97 | 0.44 | 0.20 | 0.22 |
| rs10421249 | 14384849 | CD97 | 0.59 | 0.40 | 0.56 |
| rs12973667 | 14394936 | CD97 | 0.44 | 0.53 | 0.80 |
| rs12974314 | 14402016 | CD97 | 1.00 | NA | 0.32 |
| rs2302996 | 14381460 | CD97 | 0.65 | 0.55 | 0.82 |
| rs2302997 | 14381453 | CD97 | 0.95 | 0.62 | 0.44 |
| rs35405275 | 14386797 | CD97 | 1.00 | NA | 0.31 |
| rs3786656 | 14395852 | CD97 | 0.85 | 0.64 | 0.87 |
| rs3826759 | 14394927 | CD97 | 0.64 | 0.97 | 0.86 |
| rs4468742 | 14398585 | CD97 | 0.55 | 0.45 | 0.64 |
| rs62122578 | 14382490 | CD97 | 1.00 | NA | 0.57 |
| rs9917022 | 14383839 | CD97 | 0.73 | 0.86 | 0.65 |
| rs10251380 | 140887095 | BRAF | 0.19 | 0.36 | 0.41 |
| rs1267638 | 140773646 | BRAF | 0.23 | 0.27 | 0.59 |
| rs2075046 | 140740187 | BRAF | 0.48 | 0.79 | 0.75 |
| rs2930322 | 140724782 | BRAF | 0.55 | 0.20 | 0.28 |
| rs3829814 | 140726457 | BRAF | 0.28 | 0.77 | 0.56 |
| rs74512895 | 140895216 | BRAF | 0.79 | **0.03** | **0.03** |
| rs75927580 | 140738523 | BRAF | 0.59 | 0.99 | 0.99 |
| rs76155189 | 140869869 | BRAF | 0.38 | 0.78 | 0.65 |
| rs76603725 | 140908994 | BRAF | 0.92 | **0.08** | **0.03** |
| rs77293714 | 140842909 | BRAF | 0.51 | 0.50 | 0.55 |
| rs77801931 | 140906887 | BRAF | 0.63 | 0.81 | 0.81 |
| rs78673840 | 140728253 | BRAF | 0.08 | 0.24 | 0.40 |
| rs9640168 | 140832469 | BRAF | 0.71 | 0.49 | 0.22 |
| rs9648696 | 140749350 | BRAF | 0.20 | 0.44 | 0.73 |
| rs1007086 | 50194072 | COL1A1 | 0.41 | 0.91 | 0.57 |
| rs1107946 | 50203629 | COL1A1 | 0.54 | 0.34 | 0.33 |
| rs2075558 | 50190224 | COL1A1 | 0.69 | 0.60 | 0.86 |
| rs2269336 | 50202995 | COL1A1 | 0.30 | **0.05** | **0.05** |
| rs2277632 | 50186542 | COL1A1 | 0.43 | 0.69 | 0.91 |
| rs2586488 | 50188065 | COL1A1 | 0.73 | 0.77 | 0.94 |
| rs35231764 | 50198128 | COL1A1 | 0.09 | 0.25 | 0.57 |
| rs75456035 | 50199031 | COL1A1 | 0.79 | 0.29 | 0.28 |
| rs1003349 | 22836454 | MMP14 | 0.14 | 0.20 | 0.36 |
| rs11622371 | 22848014 | MMP14 | 0.44 | **0.08** | 0.25 |
| rs11624302 | 22847238 | MMP14 | 0.55 | 0.28 | 0.50 |
| rs12893368 | 22842999 | MMP14 | 0.44 | 0.41 | 0.62 |
| rs17123036 | 22836094 | MMP14 | 0.25 | 0.93 | 0.21 |
| rs17884816 | 22846829 | MMP14 | 0.75 | **0.02** | **0.01** |
| rs2236302 | 22843345 | MMP14 | 0.28 | 0.78 | 0.58 |
| rs2236306 | 22843666 | MMP14 | 0.19 | 0.50 | 0.36 |
| rs2236307 | 22843714 | MMP14 | 0.80 | 0.50 | 0.67 |
| rs2269212 | 22840941 | MMP14 | 0.63 | 0.19 | 0.29 |
| rs743257 | 22846122 | MMP14 | 0.76 | 0.18 | 0.42 |
| rs2269213 | 22838062 | MMP15 | 0.69 | 0.37 | 0.62 |
| rs10430847 | 26570814 | MUC15 | 0.07 | **0.04** | **0.01** |
| rs10466440 | 26567258 | MUC15 | 0.41 | 0.97 | 0.97 |
| rs11822751 | 26573092 | MUC15 | 0.35 | 0.05 | 0.19 |
| rs15783 | 26565254 | MUC15 | 0.19 | 0.27 | 0.91 |
| rs17309195 | 26558573 | MUC15 | 0.20 | **0.01** | **0.07** |
| rs2063278 | 26570430 | MUC15 | 0.31 | 0.35 | 0.09 |
| rs2292290 | 26565308 | MUC15 | 0.71 | 0.47 | 0.46 |
| rs293972 | 26569918 | MUC15 | 0.59 | 0.37 | 0.33 |
| rs61877024 | 26563395 | MUC15 | 0.75 | 0.87 | 0.87 |
| rs61877025 | 26563397 | MUC15 | 0.83 | 0.84 | 0.84 |
| rs71480126 | 26570454 | MUC15 | 0.09 | 0.65 | 0.42 |
| rs967490 | 26573323 | MUC15 | 0.19 | 0.34 | 0.03 |
| rs293980 | 26565586 | MUC16 | 0.42 | 0.34 | 0.61 |
| rs18145921 | 245929906 | SMYD3 | 1.00 | 0.34 | 0.77 |
| rs7520453 | 246507179 | SMYD3 | 0.47 | 0.68 | 0.95 |
| rs11466347 | 41335193 | TGFB1 | 1.00 | NA | 0.56 |
| rs1800469 | 41354391 | TGFB1 | 0.85 | 0.68 | 0.25 |
| rs1989457 | 41346210 | TGFB1 | 0.92 | 0.18 | 0.18 |
| rs2241716 | 41348181 | TGFB1 | 0.54 | 0.89 | 0.89 |
| rs2317130 | 41355769 | TGFB1 | 0.19 | 0.53 | 0.29 |
| rs4803457 | 41355454 | TGFB1 | 0.63 | 0.48 | 0.67 |
| rs6508975 | 41338654 | TGFB1 | 0.76 | 1.00 | 1.00 |
| rs8105161 | 41333726 | TGFB1 | 0.68 | 0.57 | 0.85 |
| rs10482718 | 218345400 | TGFB2 | 0.66 | 0.92 | 0.75 |
| rs12058014 | 218343626 | TGFB2 | 0.57 | 0.88 | 0.98 |
| rs1418556 | 218442672 | TGFB2 | 0.81 | 0.56 | 0.84 |
| rs3737977 | 218442986 | TGFB2 | 0.55 | 0.84 | 0.45 |
| rs6604604 | 218344326 | TGFB2 | 0.05 | 0.32 | 0.51 |
| rs6683598 | 218444558 | TGFB2 | 0.66 | 0.92 | 1.00 |
| rs6691070 | 218343417 | TGFB2 | 0.49 | 0.74 | 0.95 |
| rs6704255 | 218444201 | TGFB2 | 0.09 | 0.65 | 0.42 |
| rs7550232 | 218345173 | TGFB2 | 0.16 | 0.70 | 0.89 |
| rs900 | 218441563 | TGFB2 | 0.73 | 0.70 | 0.95 |
| rs991967 | 218442109 | TGFB2 | 0.81 | 0.65 | 0.90 |
| rs909207 | 75983239 | TGFB3 | 0.79 | 0.37 | 0.51 |

HWE,Hardy-Weinberg equilibrium
