# Supplementary figures and images for: Gene Polymorphism of MUC15, MMP14, BRAF, and COL1A1 Is Associated with Capsule Formation in Hepatocellular Carcinoma
Source: Can J Gastroenterol Hepatol. 2021 Apr 28;2021:9990305. doi: 10.1155/2021/9990305 (PMC8100414; doi:10.1155/2021/9990305)

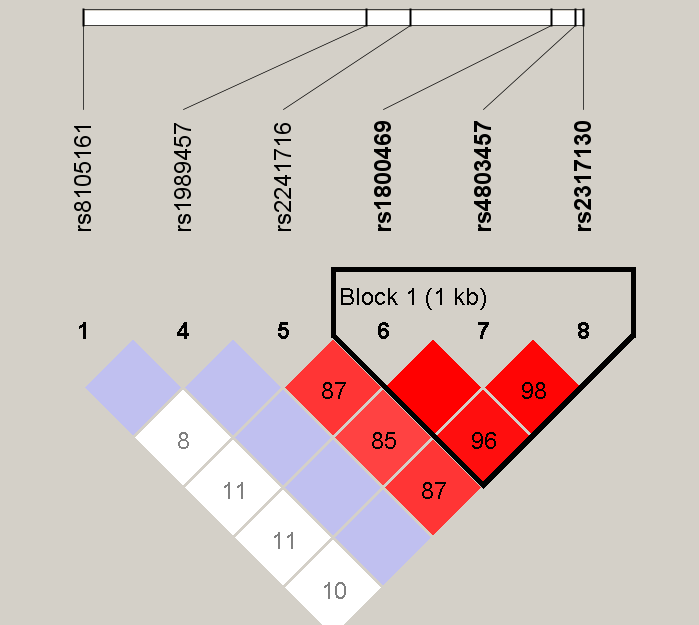


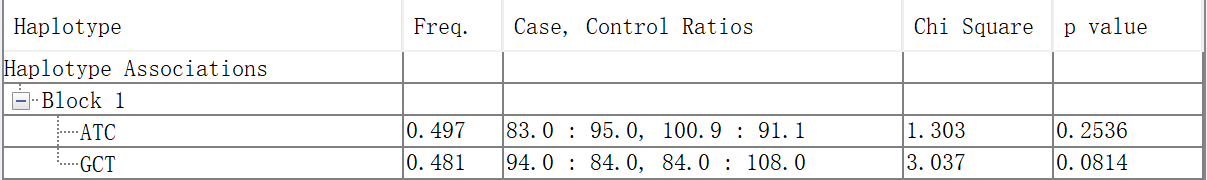


Fig.1 TGFB1 gene


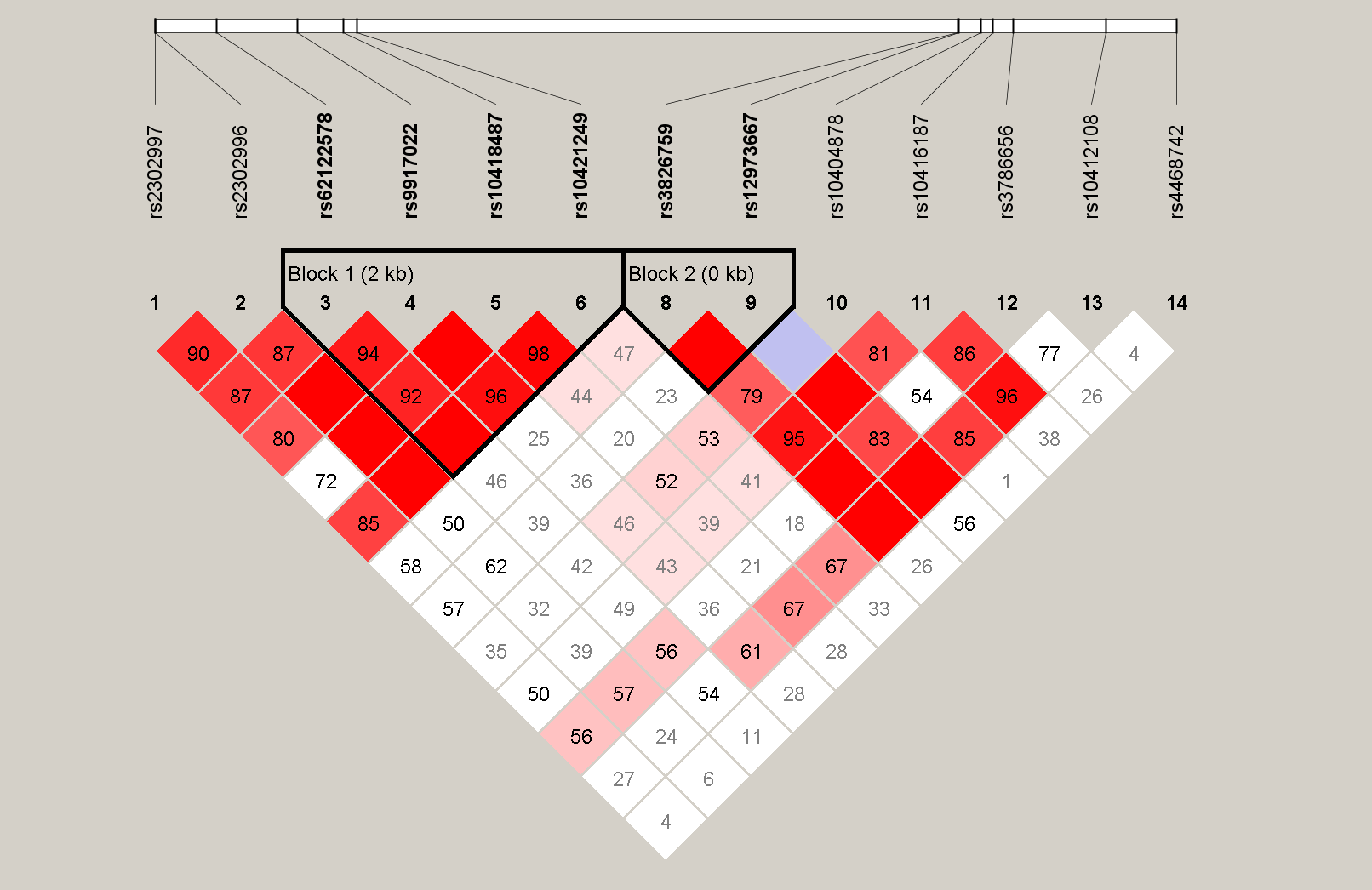


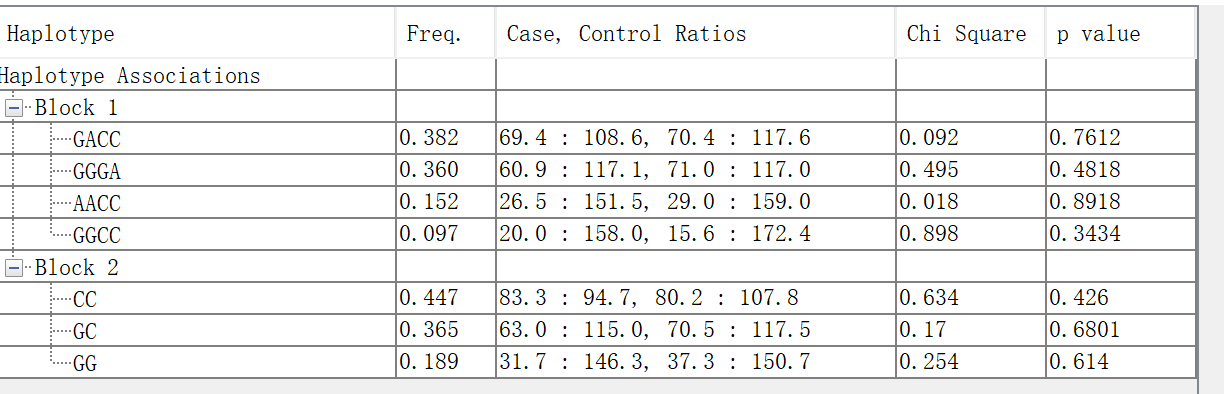


Fig.2 CD97 gene


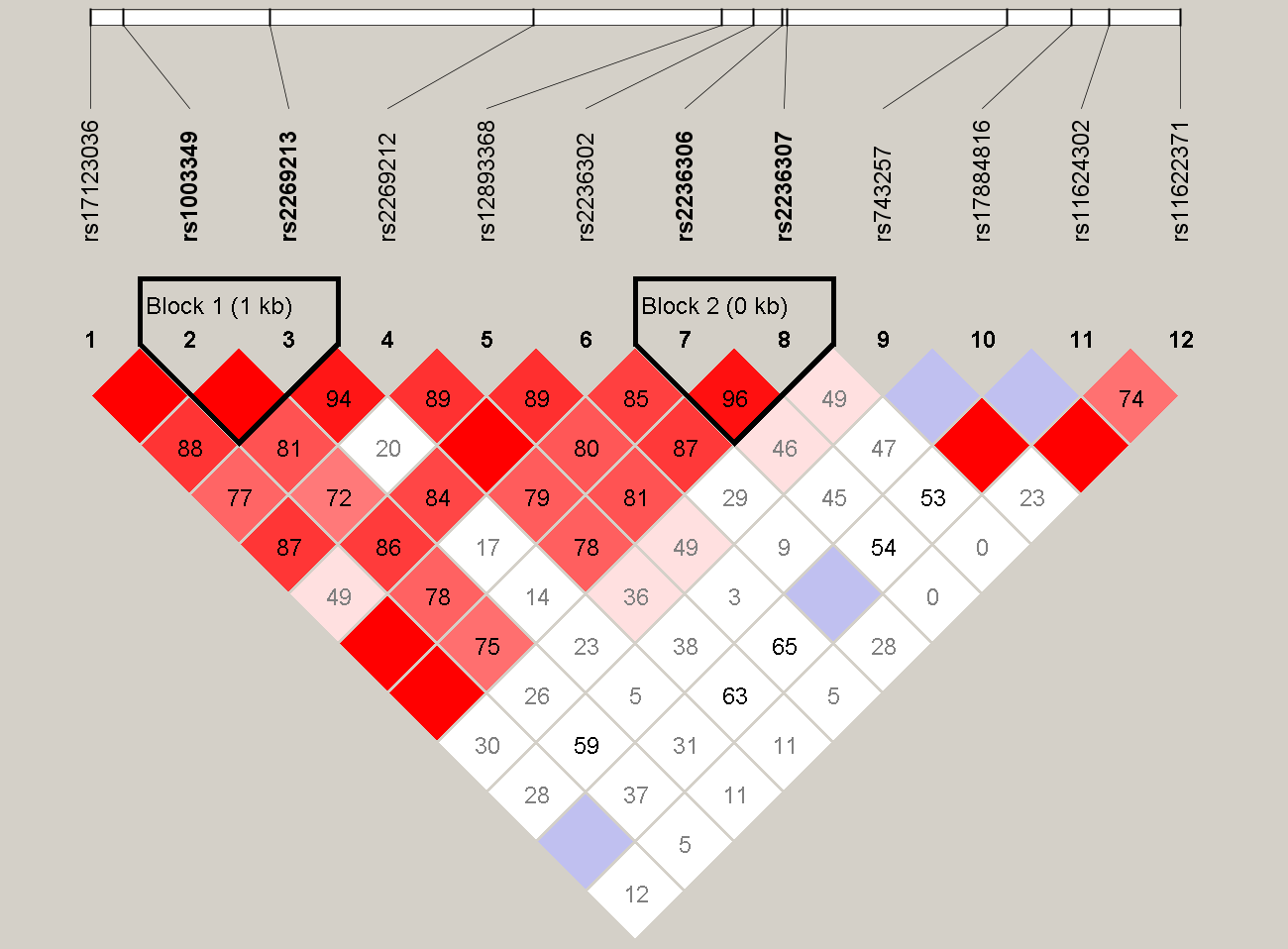


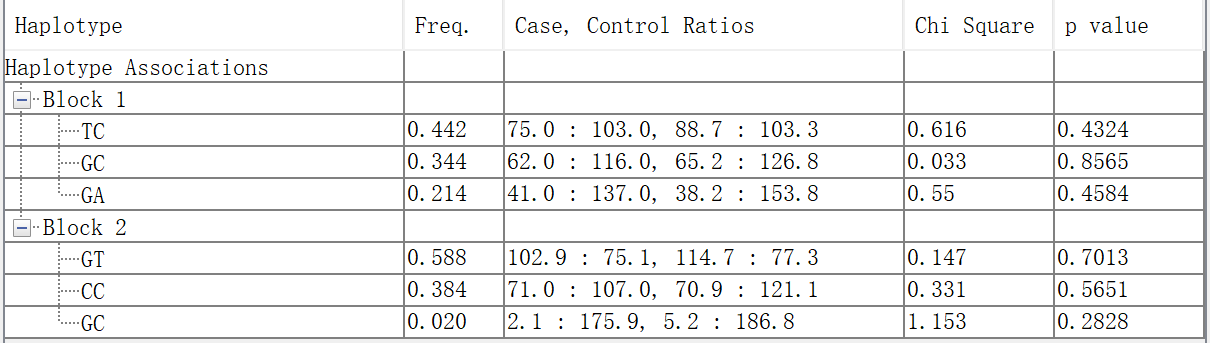


Fig.3 MMP14 gene


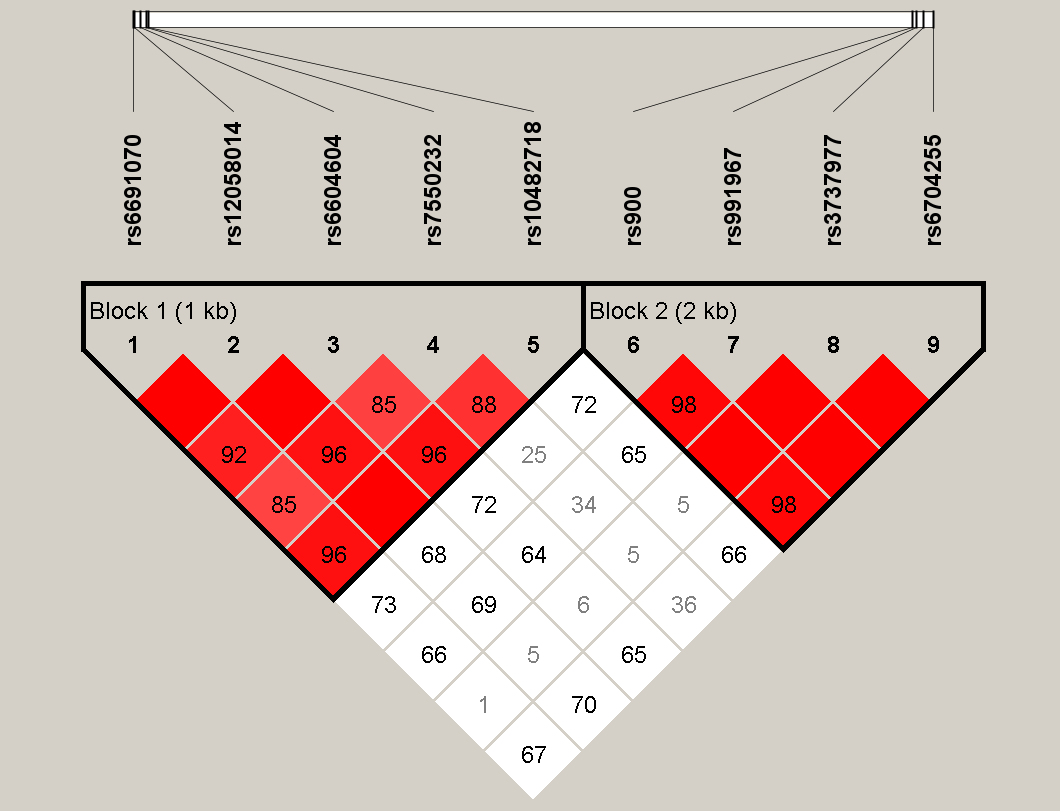


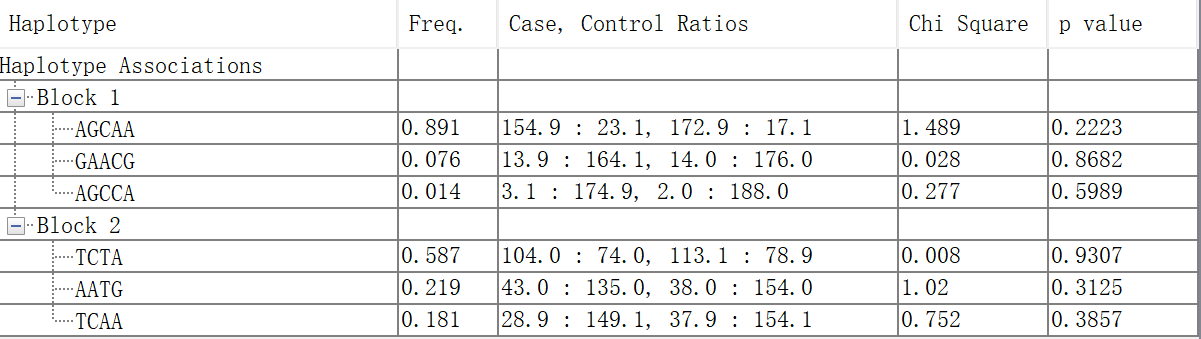


Fig.4 TGFB2 gene


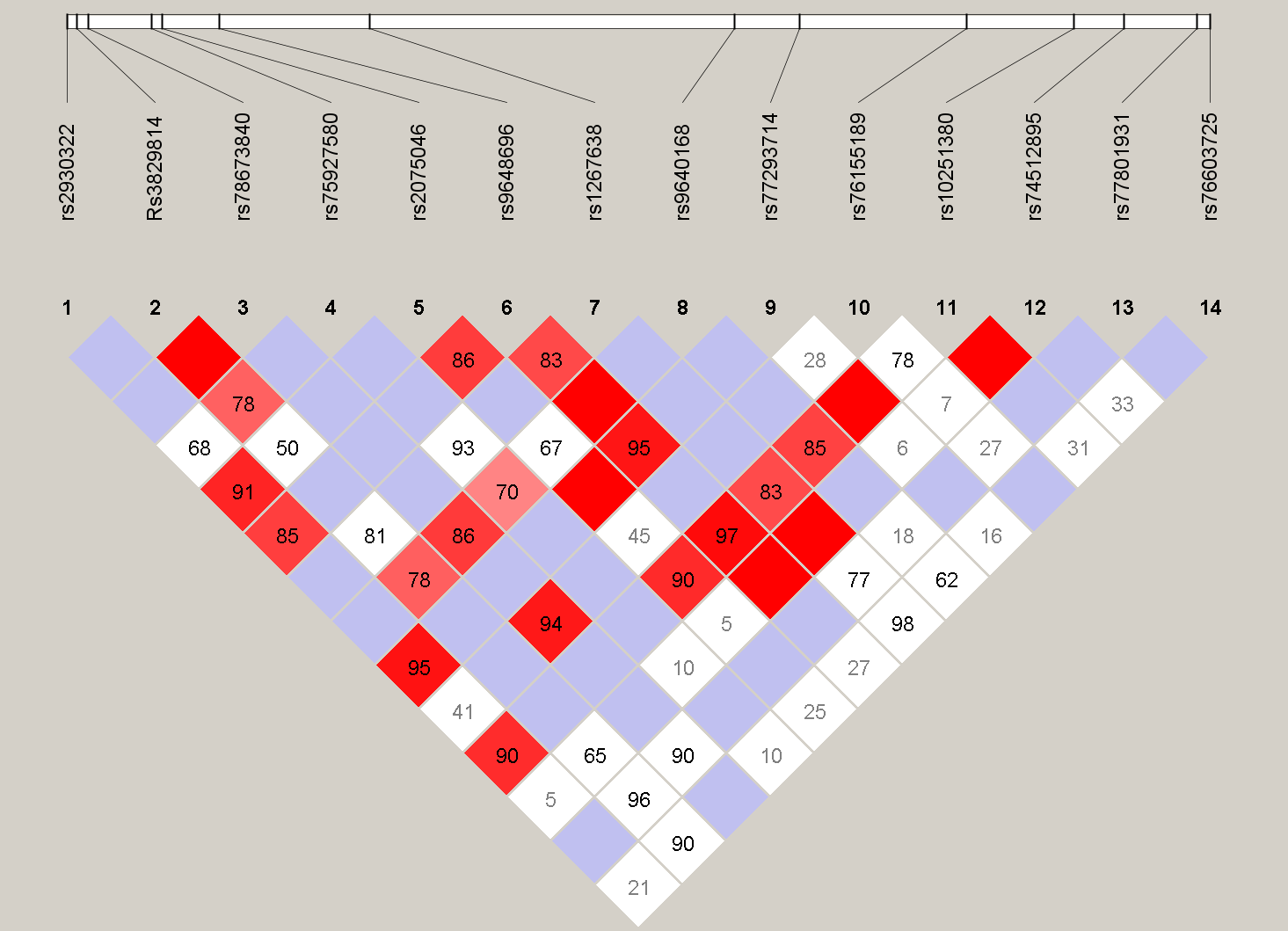


Fig.5 BRAF gene


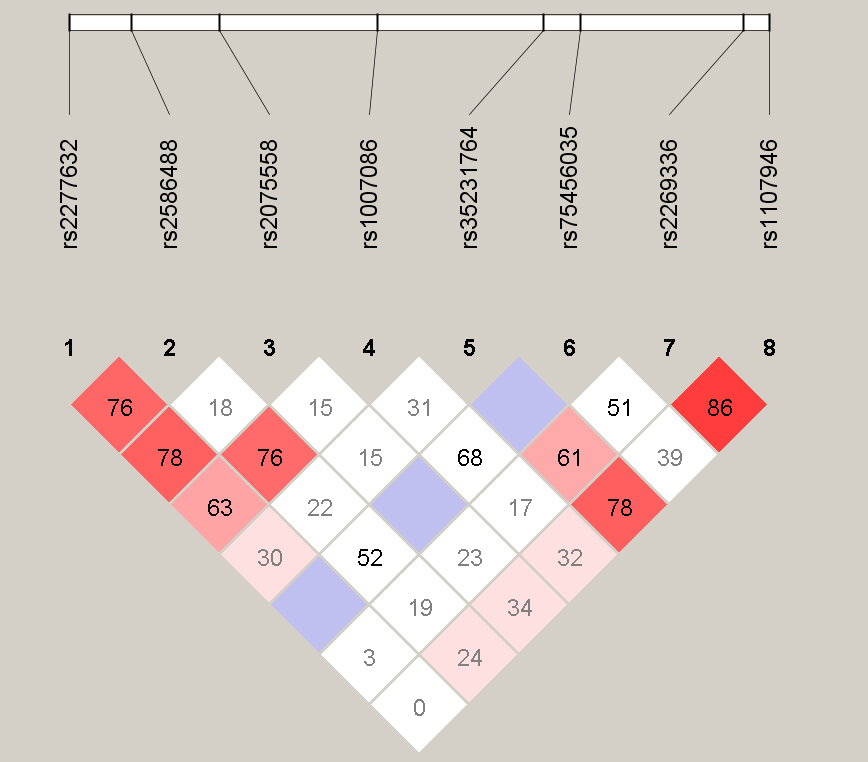


Fig.6 COLA1 gene

Supplement: Supplementary Materials — Supplementary Table S1 lists the SNP IDs, locations, and allele frequencies. Supplementary Table S2 shows that we evaluated the associations of the SNP variant genotypes with capsule formation stratified by selected variables. Supplementary Figure exhibits haplotype blocks of specific genes. [file 9990305.f1.zip › 9990305.f1/Supplementary Figure.DOCX]
